# Orthodontic management of bilateral maxillary canine-first premolar transposition and bilateral agenesis of maxillary lateral incisors: a case report

**DOI:** 10.1590/2176-9451.20.2.100-109.oar

**Published:** 2015

**Authors:** Elena Di Palma, Biagio Di Giuseppe, Michele Tepedino, Claudio Chimenti

**Affiliations:** 1 PhD, University of L'Aquila, Department of Applied Clinical Sciences and Biotechnology, L'Aquila, Italy; 2 Private practice, Coppito, L'Aquila, Italy; 3 DDS, University of L'Aquila, Department of Applied Clinical Sciences and Biotechnology, L'Aquila, Italy; 4 Professor, University of L'Aquila, Department of Applied Clinical Sciences and Biotechnology, L'Aquila, Italy

**Keywords:** Cuspid, Bicuspid, Ectopic tooth eruption, Corrective Orthodontics

## Abstract

**INTRODUCTION:**

Maxillary canine-first premolar transposition (Mx.C.P1) is an uncommon dental positional anomaly that may create many orthodontic problems from both esthetic and functional points of view.

**OBJECTIVE:**

In this report we show the orthodontic management of a case of Mx.C.P1 associated with bilateral maxillary lateral incisor agenesis and unilateral mandibular second premolar agenesis

**METHODS:**

The patient was treated with a multibracket appliance and the extraction of the lower premolar.

**RESULTS:**

treatment was completed without the need for any prosthetic replacement.

## Abstract (pt)

**INTRODUÇÃO:**

a transposição entre canino e primeiro pré-molar superiores (Mx.C.P1) é uma anomalia de posição dentária rara, que pode causar muitos problemas ortodônticos, não só com relação à estética, mas também com relação à função do paciente.

**OBJETIVO:**

no presente artigo, relatamos o manejo ortodôntico de um caso de Mx.C.P1 associada à agenesia bilateral de incisivos laterais superiores e agenesia unilateral do segundo pré-molar inferior.

**MÉTODOS:**

o paciente foi tratado com aparelho ortodôntico fixo e extração do pré-molar inferior.

**RESULTADOS:**

o tratamento foi concluído sem a necessidade de reposição protética.

## INTRODUCTION

Dental transposition is an uncommon dental anomaly involving a positional interchange of two teeth.^1^

Recent meta-analysis^2^ underlined that tooth transposition is a rare phenomenon (0.33%) with various, sometimes inexplicable, forms of manifestation and that its occurrence seems to have no specific sex predilection, but some maxillary predisposition is noted. Unilateral occurrence is considerably higher than the bilateral, but no left or right-side predilection in the maxilla or mandible has been evident. In contrast, other authors found that tooth transposition occurred more frequently in the maxillary left side.^1^^,^ 3

The most common form of transposition is between maxillary canine and first premolar (Mx.C.P1).^4^

Dental transposition represents a multifactorial condition, both genetic^1^^,^ 5 ^-^ 10 and environmental^1^^,^ 3 ^,^ 11 ^,^ 12 ^,^ 13 factors seem to be involved in the etiology of transposition.

A recent study conducted by Ely et al^6^ underlined that large-scale population-based studies will be required to further refine our understanding of the genetics of this anomaly.

Although in the literature there are several reports of maxillary canine and first premolar transpositions solved with correction of the transposition,^14^^-^ 17 this would not always be advisable from a cost-benefit point of view.^17^ In fact, when the teeth involved in the transposition are fully erupted and completely or almost completely aligned in the transposed position, a satisfactory result can be obtained by maintaining the transposition.^18^^-^ 21 In this context, iatrogenic damage to teeth and periodontal tissues can be avoided.

In this report, it is shown the orthodontic management of a case of bilateral maxillary canine-first premolar transposition (Mx.C.P1) associated with bilateral maxillary lateral incisor agenesis and unilateral mandibular second premolar agenesis.

## CASE REPORT AND DIAGNOSIS

**Figure 1 -:**
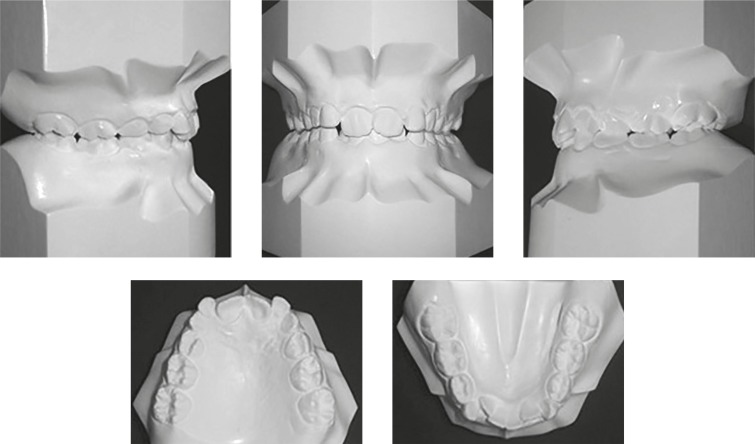
Pre-treatment dental casts.

**Figure 2 -:**
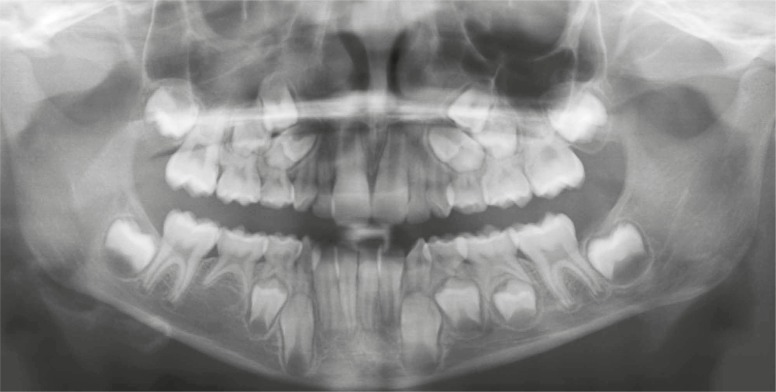
Initial panoramic radiograph showing bilateral maxillary permanent lateral incisors and second right lower premolar agenesis, and initial bilateral transposition of upper canines and first premolars.

**Figure 3 -:**
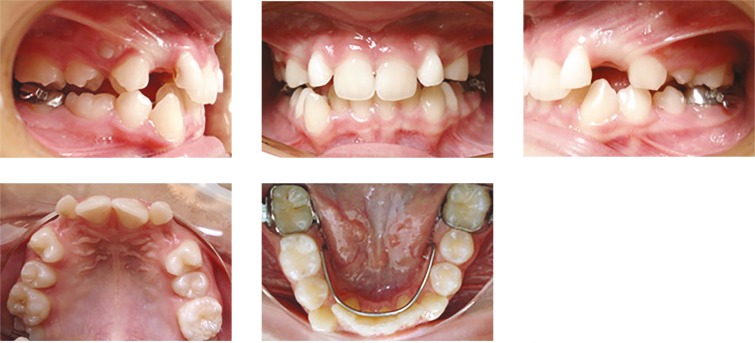
Pre-treatment intraoral views.

**Figure 4 -:**
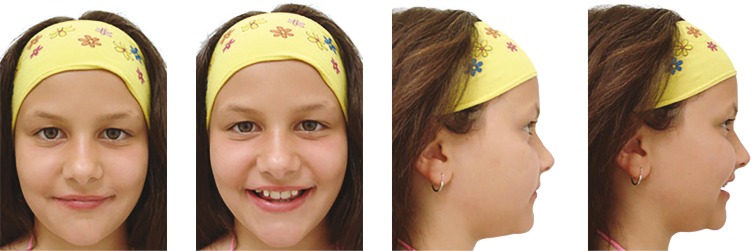
Pre-treatment facial photographs.

**Figure 5 -:**
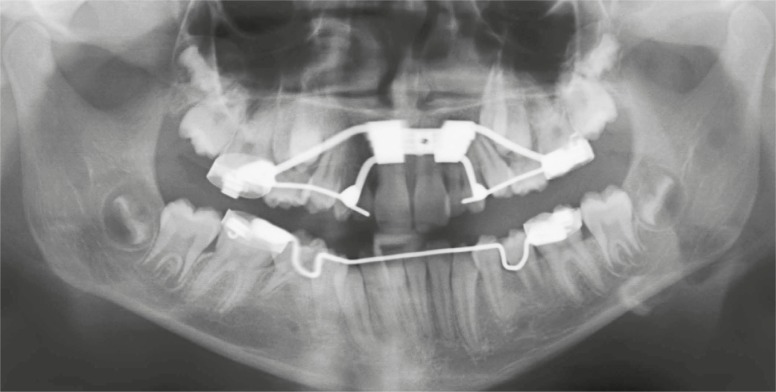
Pre-treatment panoramic radiograph taken the very moment the patient came to us for the second time. The patient was wearing a lingual arch and a rapid palatal expander. The radiograph shows bilateral maxillary permanent lateral incisors and second right lower premolar agenesis, bilateral upper lateral incisors and right second molar deciduous persistence, complete bilateral transposition of upper canines and first premolars, normal periodontal support and healthy bone.

**Figure 6 -:**
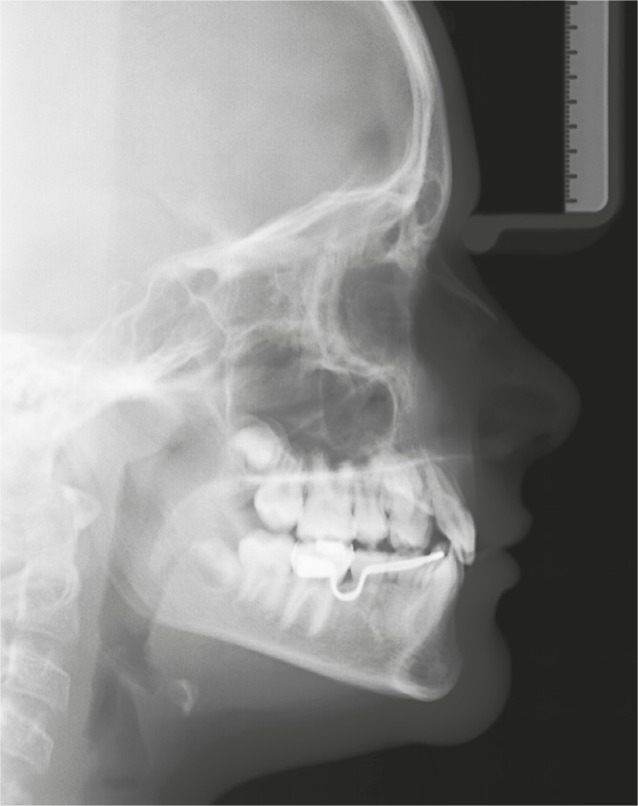
Pre-treatment cephalogram.

**Figure 7 -:**
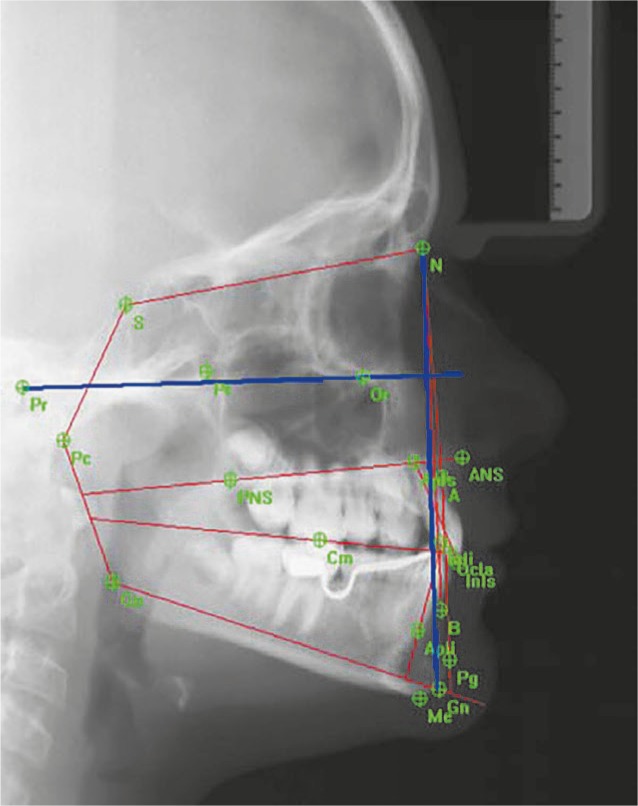
Pre-treatment cephalometric tracings.

The patient came to our observation for the first time at the age of 7 years and 6 months old (Figs 1 and 2). After that, she was treated for 2 years by another orthodontist; and later she decided to refer to us again, at the age of 10. Pre-treatment records (Figs 3-7) were taken, with previous appliances worn.

Analysis of complete diagnostic records revealed Class II division 2 malocclusion, a flat profile with bimaxillary retrusion, the mandibular arch with moderate crowding and the retention of primary right second molar. In the maxillary arch, there was retention of primary lateral incisors and the right and left canine were erupting between first and second premolars (Fig 3). The patient also presented regular oral hygiene and healthy periodontal tissues.

She showed a straight profile with bimaxillary retrusion, symmetrical frontal view and normal anterior facial height (Fig 4).

A panoramic radiograph showed bilateral maxillary permanent lateral incisors and mandibular second premolar agenesis, in addition to the bilateral transposition of canines and first premolars (Fig 5).

Cephalometric analysis (Figs 6 and 7) did not reveal any notable deviation in the skeletal and dental patterns, as shown in Table 1: skeletal Class I relationship, horizontal growth tendency and lingual inclination of mandibular incisors.

**Table 1 -:** Summary of cephalometric analysis (MP= mandibular plane; FHP= Frankfort horizontal plane; PP= palatal plane; L1= lower incisor; U1= upper incisor).

Variables	Pre-treatment	Post-treatment
	(10 years and 0 months old)	(13 years and 2 months old)
SNA (degree)	84.09	84.01
SNB (degree)	82.17	83.39
ANB (degree)	1.92	0.62
GoGn/Sn (degree)	28.85	24.23
MP/FHP (degree)	19.89	15.73
PP/MP (degree)	13.58	21.26
L1 to MP (degree)	86.52	95.68
U1 to PP (degree)	108.16	108.96

## TREATMENT

## Problems list

» Agenesis of left and right maxillary lateral incisors and right mandibular second premolar.
» Transposition of right and left maxillary canine.
» Lingual tipping of mandibular incisors.
» Moderate crowding.
» Angle Class II Division 2 malocclusion.

## Treatment options

This case can be solved in different ways:

1) Considering patient's straight profile, it would be better to maintain the spaces of lateral incisors; this treatment option requires distalization of maxillary molars to correct the Class II molar relationship and to gain the spaces needed to place endosseous dental implants. Regarding the transposition:

(1a) The ideal treatment would be to correct transposition due to functional problems related to the presence of the palatal cusp of the first premolar. The disadvantages of this approach included a long treatment period and the risk of root resorption, loss of pulp vitality or loss of hard and soft tissues of adjacent teeth.

(1b) Leaving the transposition has some disadvantages related to differences in size, shape, and tooth color between canine and premolar, which can sometimes cause esthetic problems. The gingival contour of the premolar is lower in respect to the canine, and this may require a periodontal recontouring procedure. However, even if these esthetic problems are overcome, the palatal cusp of the transposed premolar might cause functional interference, despite the control of its angulations, torques, and even after coronal reshaping. Prosthetic restoration after pulpectomy will also be necessary, if the size and shape of premolar are completely recontoured, in order to make it more similar to a canine.

In both cases, the space for an endosseous implant, in position 4.5 in the lower arch, must be kept.

2) The second choice, accepted by the patient and the parents, was not to correct the transposition and to move the maxillary first premolars into the spaces of lateral incisors. The disadvantages of this approach were esthetics and included the different color, shape and gingival contour of premolars in comparison to lateral incisors. Also, a balancing interference can occur between the palatal cusp of the premolar and the mandibular canine, thus occlusal balance is often required in order to improve function.^18^

An accurate diagnostic and interdisciplinary approach is necessary to obtain improved, conservative and predictable esthetic results in an extremely esthetical area, such as the anterior maxillary dentition.

## Treatment plan

Treatment objectives were (1) in the mandibular arch, extract the second right primary molar and the left premolar to balance the number of upper and lower teeth and to establish a correct Class I molar relationship; (2) in the maxillary arch, keep the complete bilateral transposition and replace missing maxillary lateral incisors by moving premolars mesially using a multibracket appliance; (3) establish a Class I molar and canines relationships, maintaining an ideal overjet and overbite; (4) correct lingual inclination of mandibular incisors, while maintaining the actual position of maxillary incisors; (5) maintain upper first premolars in an ideal position to obtain good conservative and esthetic restoration; (6) maintain facial balance.

## Treatment progress

In the initial phases of treatment, in the maxillary arch, 0.018-inch stainless steel sectional archwires from first molars to first premolars were used, and open coil springs were positioned between first and second premolars to facilitate eruption of canines. Lingual arch was not removed from the lower arch (Fig 3).

When maxillary canines were completely erupted, all maxillary and mandibular teeth were bonded with a multibracket appliance after removal of upper and lower primary teeth and left mandibular second premolar. On mandibular first molars, composite shims were positioned to avoid interferences in occlusion. During this phase of treatment, maxillary and mandibular 0.014-inch superelastic nickel-titanium archwires were used.

**Figure 8 -:**
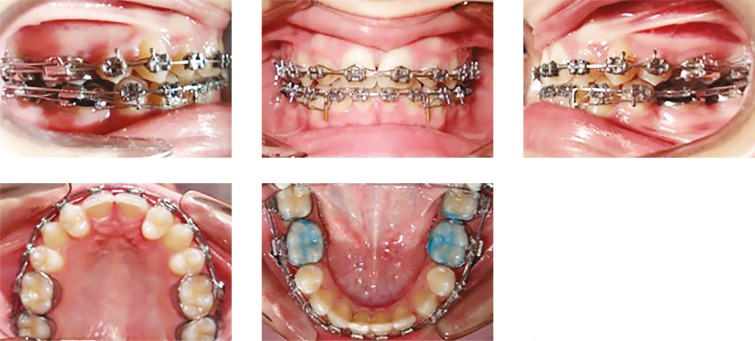
In treatment intraoral views.

**Figure 9 -:**
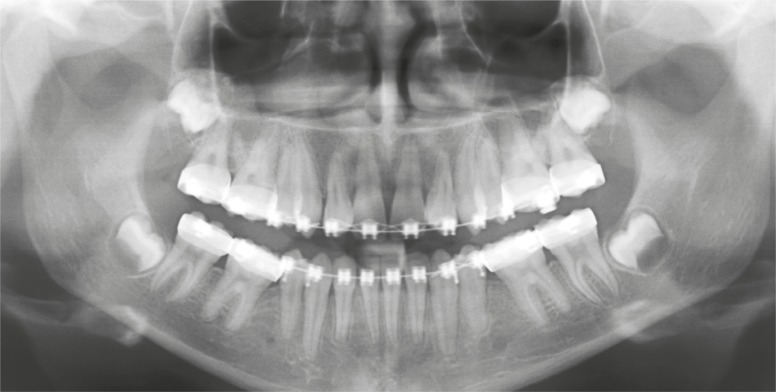
Panoramic radiograph taken during treatment.

In the final phase of treatment, 0.019 x 0.025-inch stainless steel archwires were used (Fig 8) and a panoramic radiograph was taken to assess correct root parallelism (Fig 9).

**Figure 10 -:**
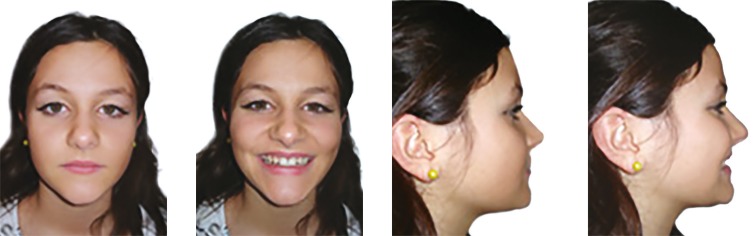
Final facial photographs.

**Figure 11 -:**
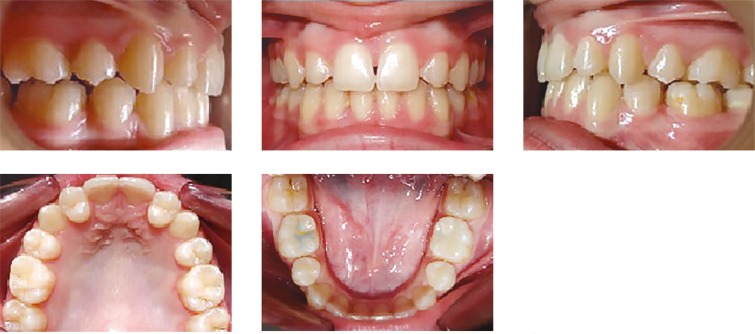
Final intraoral views.

**Figure 12 -:**
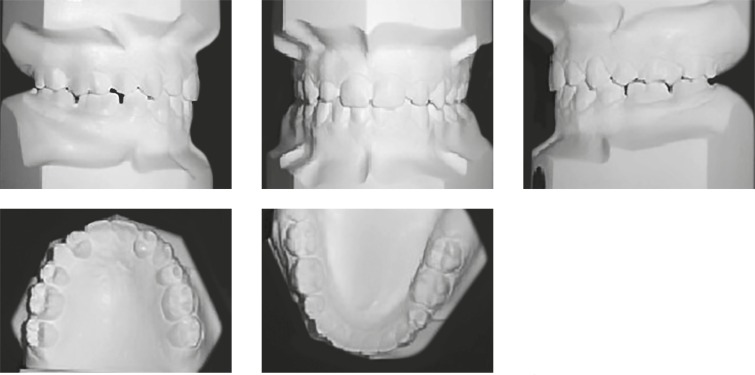
Final dental casts.

**Figure 13 -:**
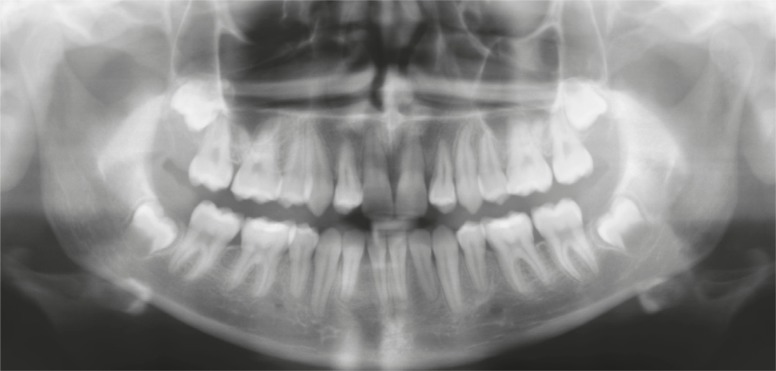
Final panoramic radiograph

**Figure 14 -:**
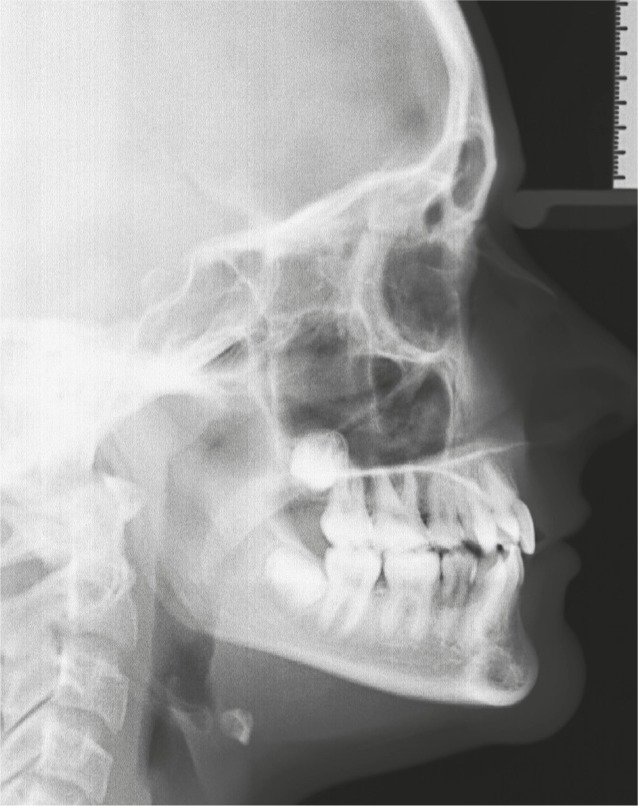
Final cephalogram.

**Figure 15 -:**
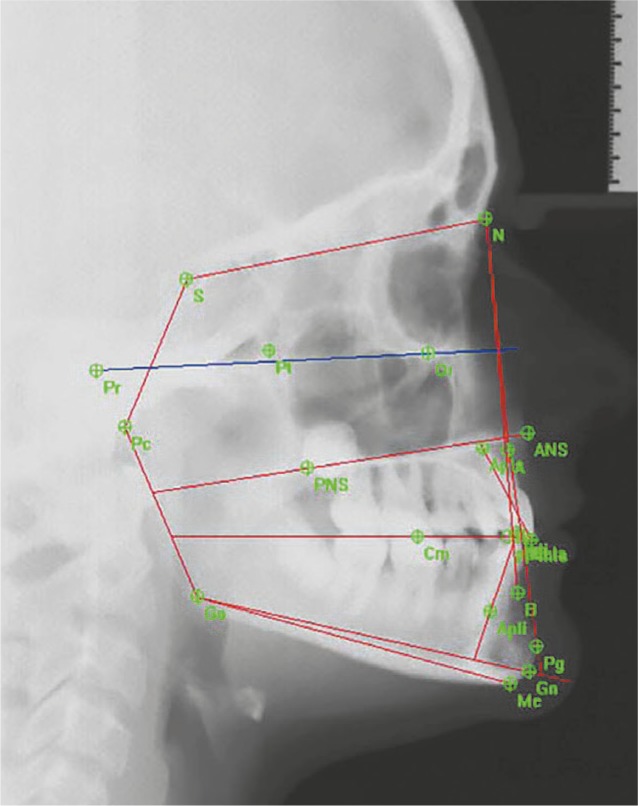
Final cephalometric tracings.

After 30 months of active treatment, the fixed appliance was removed; maxillary and mandibular removable contentions were placed for retention. Final radiographic and photographic records were taken (Figs 10-14) and an end-treatment cephalometric analysis was performed (Fig 15) in order to check whether treatment objectives were achieved.

## Treatment results

Crowding of the lower arch was corrected, a Class I molar and canine relationship was obtained as well as a good overjet and overbite (Figs 10 and 11). Lingual inclination of lower incisors was corrected (L1 to MP angle increased from 86.52° to 95.68°), the initial position of upper incisors (U1 to PP angle increased from 108.16° to 108.96°) and facial balance was maintained, as can be seen in Table 1, post-treatment cephalometric tracings (Fig 15) and extraoral photographs (Fig 10). Good root parallelism was achieved (Fig 13). Upper first premolars are well positioned and with good conservative and esthetical restoration. A beautiful and functional result will be achieved.

**Figure 16 -:**
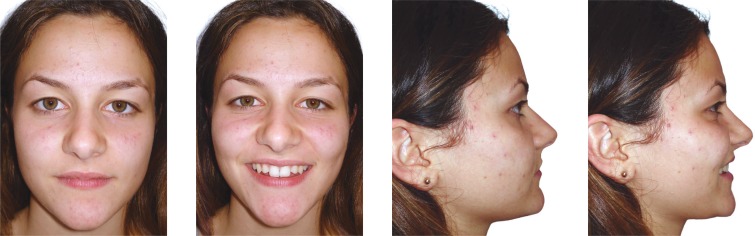
Two-year follow-up facial photographs.

**Figure 17 -:**
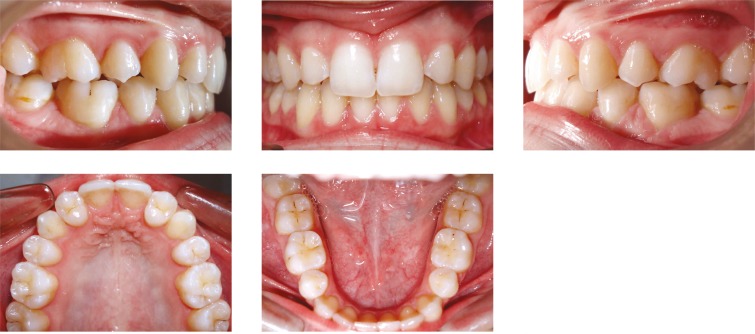
Two-year follow-up intraoral photograph

## DISCUSSION AND CONCLUSION

In several studies, it has been reported that transposed teeth are associated with dental anomalies, such as peg-shaped and congenitally missing teeth; in particular, a high incidence of congenitally missing teeth and peg-shaped lateral incisors are associated with Mx.C.P1.^1^^,^ 3 ^,^ 7 ^,^ 10 ^,^ 11 ^,^ 19

Several cases of Mx.C.P1 reported in literature are solved with the correction of transposition;^14^^-^ 17 ^,^ 22 however, this approach requires longer treatment time and stability, and esthetic and function of end results are not always granted.

In the literature, there are also many cases of transposed teeth that have been treated without the correction of transposition, and cases in which congenitally missing upper lateral incisors were substituted with the upper first premolar.^18^^,^ 19 ^,^ 23 Nestel and Walsh^18 ^reported a case of bilateral Mx.C.P1 associated with agenesis of left maxillary lateral incisor, solved maintaining the transposition in the left side and moving the premolar into the space of the missing incisor. The authors achieved good esthetic and functional results. Parker^23^ reported a very interesting case of bilateral Mx.C.P1 associated with bilateral agenesis of maxillary lateral incisors, also treated by means of maintaining the transposition and closing the spaces. Parker provided a 35-year follow-up which demonstrated that such result could be functionally and esthetically stable over time.

In the present case report, the chief complain for the patient and her parents was to achieve a definitive solution. In fact, the decision to keep the spaces of upper lateral incisors required to temporarily replace missing incisors until final prosthesis placement was possible. There is also the probability that any fixed prosthetic device will require periodical repair or replacement throughout patient's lifetime.

After having appraised the case difficulty, timing, risks, esthetics, function, stability, biological cost or damage, it was decided not to correct the transposition and to close the spaces of upper lateral incisors by moving mesially upper first premolars. Other advantages of this type of therapeutic solution are the possibility to create a canine guidance during lateral movement of the mandible and to obtain a Class I canine relationship. In addition, the size and color of maxillary premolars were very similar to that of lateral incisors. Assessment of protrusive and lateral mandibular movements reveals that, in this patient, there is no functional interference due to the palatal cusps of the transposed premolars. Furthermore, the patient could also accept the esthetic outcome and was satisfied with alignment of maxillary anterior teeth; in fact, she decided not to proceed with the esthetical reconstruction of maxillary first premolars. This outcome has been obtained within reasonable time (three years) and without iatrogenic damages.
